# Trajectories of Mid-Life Job Insecurity and Employment: Associations with Cognitive Function in the HRS-HCAP

**DOI:** 10.1093/geroni/igaf122.4289

**Published:** 2025-12-31

**Authors:** Erika Beidelman, Xuexin Yu, Rachel Donnelly, Mateo Farina, Constance Beaufils, Amanda Sonnega, Yuan Zhang

**Affiliations:** Columbia University Mailman School of Public Health, New York, New York, United States; Columbia University Mailman School of Public Health, New York, New York, United States; Vanderbilt University, Vanderbilt University, Tennessee, United States; University of Texas at Austin, Austin, Texas, United States; King’s College London, King’s College London, England, United Kingdom; University of Michigan, Ann Arbor, Michigan, United States; Columbia University, New York, New York, United States

## Abstract

Continued workforce participation may preserve cognitive health in older age. However, many Americans face job insecurity and instability in later-life which may undermine these benefits. We examined the association of employment and perceived job insecurity trajectories (1998-2014) with domain-specific cognitive functioning (2016) among Health and Retirement Study Harmonized Cognitive Assessment Protocol participants aged < 67 years in 1998 (n = 2,618). Gender-stratified sequence analyses identified clusters of employment trajectories and adjusted linear regression models—weighted for differential retention and survival—estimated associations with standardized cognitive scores. We observed six trajectory clusters for men and women: predominantly job secure employment (reference), job secure to retirement transition, predominantly job insecure employment, predominantly self-employed, predominantly retirement, not in labor force (women only), and inconsistent employment (men only). Among women, retirement, not in the labor force, and secure-to-retirement paths were linked to lower memory (β range=-2.52 to -3.94) and executive function (β range=-2.65 to -4.00) scores compared to predominantly secure employment. For men, inconsistent employment was linked to underperformance on memory (β=-4.50, 95% CI=-7.12, -1.89), executive function (β=-5.33, 95% CI=-8.09, -2.57), language (β=-3.60, 95% CI=-6.20, -1.00), and orientation (β=-0.43, 95% CI=-0.69, -0.16) while retirement predicted lower memory (β=-3.64, 95% CI=-6.08, -1.19) scores and job insecurity predicted lower language scores (β=-3.01, 95% CI=-5.80, -0.23). Cognitive deficits were concentrated among clusters defined by unstable trajectories, including early labor force exits and inconsistent employment. As interest in extending labor force participation at older ages increases, our findings underscore the role of employment stability and security on cognitive functioning.

